# Diabetes-related cognitive impairment: Mechanisms, symptoms, and treatments

**DOI:** 10.1515/med-2024-1091

**Published:** 2025-01-13

**Authors:** Xueting Yu, Huimei He, Jie Wen, Xiuyuan Xu, Zhaojuan Ruan, Rui Hu, Fang Wang, Haibing Ju

**Affiliations:** Endocrine Department, 920th Hospital of Joint Logistics Support Force, PLA, Kunming, 650000, Yunnan, China; Executive Ward Department, 920th Hospital of Joint Logistics Support Force, PLA, Kunming, 650000, Yunnan, China; Department of Hematology, The First People’s Hospital of Yunnan Province, The Affiliated Hospital of Kunming University of Science and Technology, Kunming, 650000, Yunnan, China; Endocrine Department, 920th Hospital of Joint Logistics Support Force, PLA, No. 212 Daguan Road, Xishan District, Kunming, 650000, Yunnan, China

**Keywords:** diabetes, cognitive dysfunction, hyperglycemia, executive function, neuroprotection

## Abstract

**Background:**

Diabetes-related cognitive impairment is increasingly recognized as a significant complication, profoundly impacting patients’ quality of life. This review aims to examine the pathophysiological mechanisms, clinical manifestations, risk factors, assessment and diagnosis, management strategies, and future research directions of cognitive impairment in diabetes.

**Methodology:**

A comprehensive literature search was conducted using PubMed, Medline, and other medical databases to identify, review, and evaluate published articles on cognitive impairment in diabetes. The search focused on studies examining pathophysiology, clinical presentations, risk factors, diagnostic approaches, and management strategies.

**Results:**

The review of current literature revealed that chronic hyperglycemia, insulin resistance, and vascular factors are major contributing factors to cognitive deficits in diabetes. Clinical manifestations include impairments in attention, memory, executive function, visuospatial abilities, and language. Risk factors encompass disease duration, glycemic control, presence of complications, age, education level, and comorbidities. Assessment tools include cognitive screening instruments, neuropsychological testing, and neuroimaging techniques. Management strategies involve glycemic control optimization, lifestyle modifications, cognitive training, and pharmacological interventions.

**Conclusion:**

This review highlights the significant prevalence and impact of cognitive impairment in diabetes, resulting from complex metabolic and vascular disturbances. Early detection and multifaceted interventions are crucial for preserving cognitive function and improving patient outcomes. Future research should focus on neuroprotective strategies, biomarker identification, and personalized approaches. Collaborative efforts between clinicians and researchers are essential to effectively address this growing healthcare challenge and enhance the quality of life for individuals with diabetes-related cognitive impairment.

## 1 Introduction

Diabetes mellitus, a chronic metabolic disorder characterized by dysregulated glucose homeostasis, is a major public health concern worldwide [1]. While the traditional complications of diabetes, such as retinopathy [2], nephropathy, and cardiovascular disease, have been extensively studied [3], increasing attention is being directed toward the impact of diabetes on cognitive function and brain health. Cognitive impairment, defined as a decline in one or more cognitive domains including attention, memory, executive function, visuospatial abilities, and language, has emerged as a prevalent complication of both type 1 and type 2 diabetes [4,5,6,7]. This impairment can range from subtle deficits in specific cognitive domains to more severe forms, such as vascular dementia or Alzheimer’s disease [8]. The pathophysiology of diabetes-associated cognitive dysfunction is multifaceted, involving direct effects of chronic hyperglycemia, insulin dysregulation, vascular pathologies, and potentially other mechanisms.

Cognitive impairment in individuals with diabetes is increasingly recognized as a significant clinical concern, with far-reaching implications for patients, healthcare systems, and society as a whole. Epidemiological studies have consistently reported a striking higher prevalence of cognitive deficits and an increased risk of dementia among people with diabetes compared to those without the condition [9]. According to the International Diabetes Federation, approximately 537 million adults (20–79 years) were living with diabetes in 2021, and this number is projected to rise to 783 million by 2045 [9]. In terms of cognitive impairment, it has been estimated that individuals with diabetes have a 73% higher risk of all types of dementia, a 56% higher risk of Alzheimer’s disease, and a 127% higher risk of vascular dementia compared to individuals without diabetes. Alarmingly, the prevalence of mild cognitive impairment in diabetes has been estimated to range from 20 to 30%, while the risk of developing dementia is approximately 1.5 to 2.5 times higher in individuals with diabetes [10]. These cognitive impairments can profoundly impact various aspects of daily life, from basic functional abilities and independence to disease self-management and overall quality of life. Moreover, the consequences extend beyond the individual, placing substantial burdens on caregivers and healthcare resources, resulting in increased healthcare costs and societal economic strain.

This narrative review examines cognitive impairment in diabetes, focusing on pathophysiological mechanisms, clinical manifestations, risk factors, and current assessment and management strategies. By synthesizing existing knowledge and identifying research gaps, we aim to underscore the significance of this complication and guide future research and clinical advancements in the field.

## 2 Literature search and scrutiny

A comprehensive literature search was conducted to identify relevant studies on cognitive impairment in diabetes. The following electronic databases were searched from their inception to March 31, 2024: PubMed/MEDLINE, Embase, Web of Science, and Cochrane Library. The search strategy included a combination of Medical Subject Headings terms and keywords related to diabetes and cognitive impairment. The main search terms for diabetes included “diabetes mellitus,” “type 1 diabetes,” “type 2 diabetes,” “insulin resistance,” and “hyperglycemia.” For cognitive impairment, the terms “cognitive dysfunction,” “cognitive decline,” “dementia,” “mild cognitive impairment,” “Alzheimer’s disease,” and “vascular dementia” were used. These terms were combined using Boolean operators (AND, OR) to refine the search. Studies were included if they met the following criteria: published in English, focused on the relationship between diabetes and cognitive impairment, included human subjects, and were original research articles, systematic reviews, or meta-analyses. Studies were excluded if they were not peer-reviewed (e.g., conference abstracts, letters to the editor), focused solely on animal models, or were case reports or small case series (*n* < 10).

## 3 Pathophysiology underlying the cognitive impairment of diabetes

### 3.1 Effects of hyperglycemia on the brain

**Figure 1:**
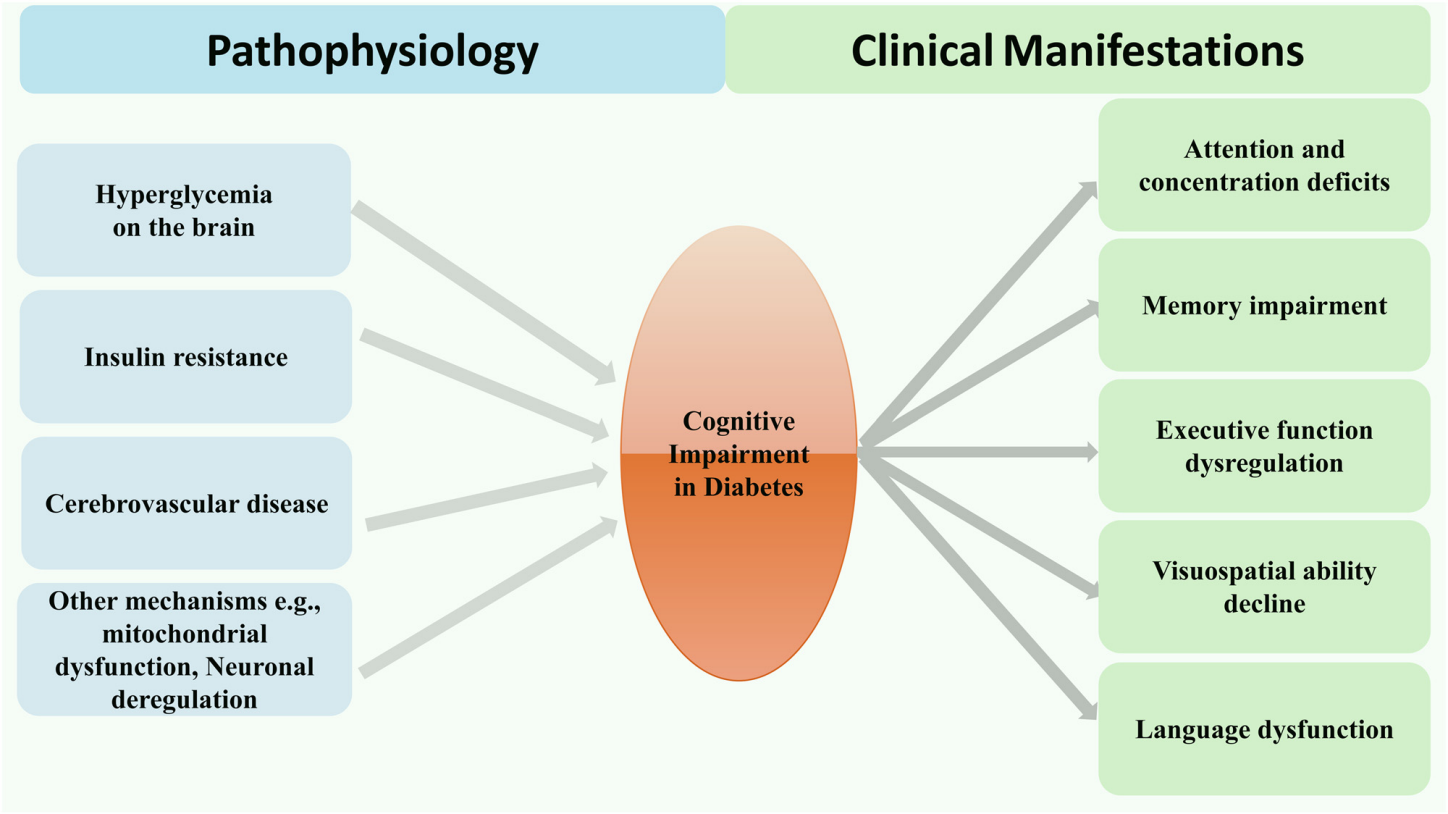
The pathophysiology and clinical manifestations of cognitive impairment in diabetic patients. The pathophysiology of cognitive impairment in diabetes involves several interconnected mechanisms. Hyperglycemia: chronic high blood glucose levels lead to oxidative stress, formation of AGEs, and disruption of the BBB, causing neuronal damage and cognitive deficits [11,12,13,14,15,16,17,18,19]. Insulin resistance and dysregulation: Impaired insulin signaling in the brain affects neuronal survival, synaptic plasticity, and neurotransmitter regulation, contributing to cognitive dysfunction [20,21,22,23,24,25,26,27]. Vascular injury: Diabetes-related vascular pathologies, including endothelial dysfunction, atherosclerosis, and cerebral hypoperfusion, can lead to ischemic brain damage and cognitive decline [28,29,30,31,32,33,34,35,36]. Neurotransmitter dysregulation: Alterations in neurotransmitter systems, such as acetylcholine, dopamine, and glutamate, affect various cognitive processes [37]. Mitochondrial dysfunction: Impaired energy metabolism and increased ROS production in neurons contribute to cognitive impairment [38]. Neuroinflammation: Chronic low-grade inflammation associated with diabetes can exacerbate neuronal damage and cognitive decline. Impaired neurogenesis and synaptic plasticity: Diabetes can disrupt the formation of new neurons and synaptic connections, crucial for learning and memory [38,39]. Epigenetic modifications: Diabetes-induced epigenetic changes can alter gene expression patterns, potentially contributing to cognitive dysfunction [40,41,42,43]. Navigation or interpreting visual information [61,62]. Cognitive impairment in diabetes primarily affects the following domains: Attention and concentration deficits [49,50,51,52,53]; memory impairment (short-term and long-term) [54,55,56,57,58]; executive function dysregulation [59,60]; visuospatial ability decline [61,62]; and language dysfunction [63,64,65,66].

Chronic hyperglycemia is a major contributor to cognitive impairment in diabetes [11] (Figure 1). It induces oxidative stress through reactive oxygen species (ROS) production [12], causing neuronal damage and cognitive deficits [13]. Hyperglycemia also accelerates the formation of advanced glycation end-products (AGEs) [14], leading to neuroinflammation and impaired insulin signaling [15,16]. It disrupts cerebral blood flow and blood–brain barrier (BBB) integrity [17], causing endothelial dysfunction, reduced cerebral perfusion [18], and increased BBB permeability, allowing neurotoxic substances to enter the brain [19].

### 3.2 Role of insulin resistance and dysregulation

Insulin functions as a critical neuromodulator in the brain, influencing neuronal survival, synaptic plasticity, and cognitive processes [20,21,22]. In diabetes, insulin resistance and dysregulation disrupt brain insulin signaling, impairing neuronal function and cognition [23,24]. This leads to decreased glucose uptake, increased oxidative stress, and impaired synaptic plasticity [1]. Chronic hyperglycemia exacerbates insulin resistance through oxidative stress, inflammation, and AGE accumulation [12,25]. Insulin dysregulation in both type 1 and 2 diabetes affects neurotransmitter systems, neuronal energy metabolism, and neurogenesis [26,27].

### 3.3 Vascular factors and cerebrovascular disease

Diabetes is a risk factor for cerebrovascular disease, impacting cognitive function [28]. Stroke can cause focal neuronal damage and cognitive impairment [29,30]. Diabetics are at increased risk of silent brain infarcts and white matter lesions [31], disrupting brain connectivity and contributing to cognitive deficits [32]. Diabetes-associated endothelial dysfunction and impaired cerebrovascular autoregulation compromise brain oxygen and nutrient delivery [33,34], leading to reduced vasoreactivity and increased ischemic injury susceptibility [35]. Vascular pathologies like atherosclerosis increase the risks of cerebral hypoperfusion and microvascular ischemic lesions, exacerbating cognitive decline [36].

### 3.4 Other potential mechanisms

In addition to the mechanisms discussed above, several other biological processes have been implicated in the development of cognitive impairment in diabetes (Figure 1):

1. Dysregulation of neurotransmitter systems: Diabetes has been associated with alterations in the levels and signaling of various neurotransmitters, such as acetylcholine, dopamine, and glutamate, which play crucial roles in cognitive processes like attention, memory, and executive function [9,37].
2. Mitochondrial dysfunction: Hyperglycemia, oxidative stress, and insulin resistance can lead to mitochondrial dysfunction in neurons, resulting in impaired energy metabolism, increased ROS production, and ultimately neuronal damage and cognitive deficits [38].
3. Impaired neurogenesis and synaptic plasticity: Diabetes has been shown to disrupt adult neurogenesis, the process of generating new neurons in specific brain regions like the hippocampus. Additionally, insulin resistance and other metabolic disturbances can impair synaptic plasticity, which is crucial for learning and memory formation [38,39].
4. Epigenetic modifications: Emerging evidence suggests that diabetes can induce epigenetic changes, such as DNA methylation and histone modifications, which can alter gene expression patterns and contribute to cognitive impairment [40,41,42,43].
5. Comorbidities: Diabetes often coexists with other conditions like depression, obesity, and sleep disturbances, which can exacerbate cognitive deficits through shared pathways or additive effects [44,45]. For example, depression has been linked to neuroinflammation, decreased neurogenesis, and altered neurotransmitter levels, all of which can impact cognitive function [46,47,48].

## 4 Clinical manifestations

### 4.1 Attention and concentration deficits

Impairments in attention and concentration are among the most commonly reported cognitive deficits in individuals with diabetes [49] (Figure 1). These deficits manifest as difficulty sustaining focused attention, increased distractibility, and reduced processing speed [50,51]. Patients may struggle to concentrate on tasks, follow conversations or instructions, and experience frequent attention lapses, affecting daily functioning and productivity. Research has shown that individuals with diabetes may exhibit deficits in specific attention components, such as divided attention (attending to multiple stimuli simultaneously) and selective attention (focusing on relevant information while filtering distractions) [52,53]. These attentional impairments significantly impact activities requiring multitasking.

### 4.2 Memory impairment

Memory deficits are a prominent feature of cognitive impairment in diabetes, affecting both short-term (working) and long-term memory [54]. Short-term memory impairments manifest as difficulty remembering instructions, names, or carrying out multi-step tasks. Long-term memory deficits can compromise the ability to recall past events, learned information, or previously acquired skills, significantly impacting daily life and independence [55,56,57]. Specific memory types, such as episodic memory (personal experiences) and semantic memory (general knowledge), may be differentially affected, with some studies suggesting more pronounced impairment in episodic memory in individuals with diabetes [58].

### 4.3 Executive function dysregulation

Executive functions, encompassing higher-order cognitive processes, are often impaired in individuals with diabetes [59]. This impairment manifests as difficulties in planning, organization, cognitive flexibility, decision-making, judgment, inhibition, and self-regulation. Individuals may struggle with formulating and executing plans, prioritizing tasks, organizing information, and adapting to changing demands. They may also exhibit impaired decision-making abilities, poor risk assessment, and challenges in evaluating the consequences of actions [59]. Additionally, difficulties in controlling impulses, regulating emotions and behavior, and resisting distractions are common. Executive dysfunction profoundly impacts the ability to navigate complex situations, solve problems, and engage in goal-directed behaviors [60]. This can lead to challenges in diabetes self-care, work responsibilities, and everyday activities.

### 4.4 Visuospatial ability decline

Individuals with diabetes may experience visuospatial deficits, affecting their perception, processing, and manipulation of visual and spatial information [61]. These deficits can manifest as impaired visual perception (difficulty recognizing objects, faces, or patterns), spatial disorientation (challenges with navigation and understanding spatial relationships), and constructional apraxia (difficulty copying geometric designs or assembling structures from visual cues) [62]. These impairments have real-world implications for tasks such as driving, reading maps, interpreting diagrams, and activities requiring visual-motor coordination like dressing, cooking, or using tools. Awareness of these potential impacts and seeking appropriate support and strategies are important for the effective management of visuospatial deficits in individuals with diabetes.

### 4.5 Language dysfunction

Language deficits, though less commonly reported, are significant in individuals with diabetes [63]. These can manifest as word-finding difficulties, comprehension deficits, and fluency disturbances [64,65]. Word-finding difficulties involve challenges in recalling specific words or names. Comprehension deficits may lead to difficulties in understanding complex information or following instructions. Fluency disturbances can cause disruptions in speech flow, with hesitations or disorganized patterns. These language deficits, often subtle, can interfere with effective communication, comprehension of complex information, and self-expression, potentially impacting social interactions, educational or occupational performance, and quality of life [66].

## 5 Risk factors

### 5.1 Diabetes-related factors (duration, glycemic control, complications)

Longer disease duration in diabetes is consistently associated with a higher risk of cognitive impairment [67]. Prolonged exposure to chronic hyperglycemia, oxidative stress, and metabolic disturbances can lead to progressive neuronal damage and cognitive decline [68]. Poor glycemic control, indicated by elevated glycated hemoglobin levels, correlates with more significant cognitive deficits [69]. Sustained hyperglycemia exacerbates oxidative stress, inflammation, and vascular dysfunction, contributing to cognitive impairment [51]. Other diabetes complications, such as retinopathy, nephropathy, and cardiovascular disease, further increase the likelihood of cognitive impairment [70]. These complications share common mechanisms like microvascular and macrovascular damage, leading to neuronal injury and cognitive deficits [71]. Effectively managing disease duration, glycemic control, and complications is crucial for minimizing diabetes’ impact on cognitive function.

### 5.2 Demographic factors (age, education, socioeconomic status [SES])

Increasing age is a well-established risk factor for cognitive decline in individuals with diabetes. The aging brain is more susceptible to hyperglycemia, oxidative stress, and vascular damage, potentially accelerating cognitive impairment [72]. Lower educational attainment is associated with a higher risk of cognitive impairment in diabetics. Education is believed to build cognitive reserve, potentially mitigating neuronal damage and delaying cognitive deficits [73]. Furthermore, individuals with diabetes and low SES are at increased risk of cognitive impairment [74,75]. Those with low SES may face barriers to quality healthcare, struggle with treatment adherence, and have a higher burden of other health conditions, all potentially contributing to long-term cognitive decline [74,75].

### 5.3 Comorbidities (hypertension, dyslipidemia, depression)

Elevated blood pressure in diabetes increases cognitive impairment risk by exacerbating vascular damage, cerebral hypoperfusion, and cerebrovascular lesions [76]. Abnormal lipid profiles, including high low-density lipoprotein cholesterol and triglycerides, also increase this risk [77]. Dyslipidemia leads to atherosclerosis, endothelial dysfunction, and cerebrovascular disease, affecting brain blood flow and nutrient delivery. Depression, often coexisting with diabetes, exacerbates cognitive deficits through neuroinflammation, neurotransmitter dysregulation, and decreased neurogenesis [78,79]. Managing hypertension, dyslipidemia, and depression is crucial for preventing cognitive impairment and promoting brain health in diabetics.

### 5.4 Lifestyle factors (diet, physical activity, smoking)

Unhealthy dietary patterns, high in refined carbohydrates, saturated fats, and ultra-processed foods increase cognitive impairment risk in diabetics by promoting oxidative stress, inflammation, and metabolic dysregulation [80]. Regular physical activity has protective effects, improving glycemic control, reducing inflammation, and stimulating neurogenesis and brain plasticity [81]. Cigarette smoking significantly increases cognitive decline risk in diabetics by exacerbating oxidative stress, inflammation, and vascular damage [82]. Prioritizing a balanced diet, incorporating regular exercise, and smoking cessation are crucial strategies for preserving cognitive function in individuals with diabetes.

By identifying and addressing these modifiable risk factors, healthcare professionals can implement targeted interventions to mitigate the risk of cognitive impairment in individuals with diabetes, ultimately improving cognitive outcomes and quality of life.

## 6 Diagnosis, assessment, and management

### 6.1 Cognitive assessment approaches

**Figure 2:**
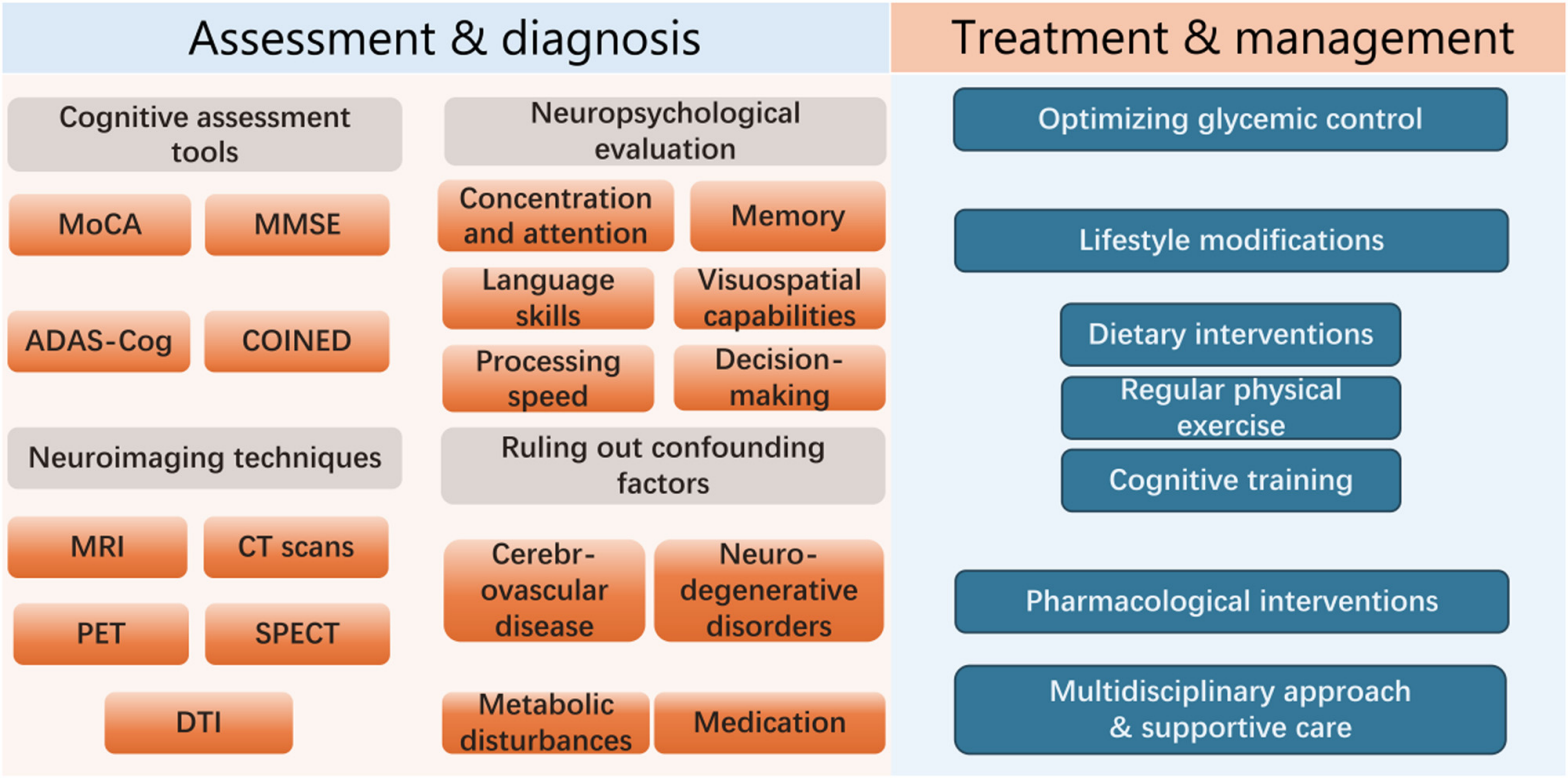
Assessment and diagnosis approaches for cognitive impairment in diabetic patients and the management strategies. Key research achievements in the field of diabetes and cognitive dysfunction include establishing the link between diabetes and increased risk of cognitive impairment and dementia [9,10], pinpointing risk factors including disease duration, glycemic control, and comorbidities [67,68,69,70,71,72,73,74,75,76,77,78,79,80,81,82], developing assessment tools like cognitive screening and neuroimaging techniques [83,84,85,86,87,88], and exploring management strategies such as glycemic control optimization, lifestyle modifications, and potential pharmacological interventions [91,92,93,94,95,96,97,98,99,100,101,102,103,104,105,106,107,108].

Cognitive screening tools play a vital role in detecting cognitive impairments and determining the need for further evaluation, especially in clinical settings. These tools help healthcare professionals identify potential issues early on and take appropriate actions. Among the commonly used cognitive screening tools in diabetes are the Montreal cognitive assessment, mini-mental state examination, Alzheimer’s disease assessment scale-cognitive subscale, and psychometrics and cognitive impairment screening in diabetes [83] (Figure 2). While cognitive screening tools provide a quick assessment of cognitive function, they are not diagnostic instruments [84]. Positive screening results should prompt further evaluation using comprehensive neuropsychological testing. Neuropsychological evaluation consists of a thorough assessment of cognitive functions utilizing standardized assessments administered by trained neuropsychologists, which covers a variety of cognitive areas, including concentration and attention, memory (verbal, visual, short term, long term), functions related to executive tasks (such as planning, problem-solving, and cognitive flexibility), language skills (naming, fluency, comprehension), visuospatial capabilities (perception, construction, spatial reasoning), and processing speed [85]. Through neuropsychological testing, a detailed profile of an individual’s cognitive strengths and weaknesses can be obtained for creating tailored interventions and tracking cognitive alterations over the long term.

Neuroimaging techniques, such as magnetic resonance imaging and computed tomography scans, offer a valuable means of understanding both the structural and functional changes within the brain that are linked to cognitive impairment [86]. These imaging modalities can detect macrostructural changes like brain atrophy, white matter lesions, infarcts, and other abnormalities that may play a role in cognitive deficits [86,87]. Additionally, functional imaging techniques like functional magnetic resonance imaging, positron emission tomography, and single-photon emission computed tomography provide important information on brain activity and metabolism, offering insights into regional brain function and neural network connectivity. This can help pinpoint areas of altered brain function that are associated with cognitive impairment. Furthermore, diffusion tensor imaging enables the evaluation of the integrity of white matter tracts in the brain, which are essential for efficient communication between different brain regions [88]. Neuroimaging findings can complement cognitive and neuropsychological assessments by shedding light on the underlying pathophysiology of cognitive impairment in individuals with diabetes.

When evaluating cognitive impairment in individuals with diabetes, it is essential to carefully consider and exclude other possible factors and comorbidities. Key assessments include evaluating vascular cognitive impairment by examining cerebrovascular diseases such as stroke or white matter lesions [89], differentiating diabetes-related cognitive impairment from neurodegenerative disorders like Alzheimer’s disease or Lewy body dementia, reviewing medications for potential cognitive side effects, and evaluating metabolic disturbances such as hypoglycemia, hypothyroidism, or vitamin deficiencies [90]. Thorough assessment and ruling out confounding factors enables healthcare professionals to accurately identify and characterize cognitive impairment in individuals with diabetes, facilitating the implementation of appropriate interventions and management strategies.

### 6.2 Treatment and management strategies

#### 6.2.1 Glycemic control

Achieving and maintaining optimal glycemic control is crucial in managing diabetes-related cognitive impairment, as chronic hyperglycemia contributes to oxidative stress, neuroinflammation, and neuronal injury [91]. Intensive glycemic control through lifestyle modifications, oral hypoglycemic agents, and/or insulin therapy can improve cognitive function and slow cognitive decline in diabetics [92] (Table 1). The diabetes control and complications trial and its follow-up, the epidemiology of diabetes interventions and complications, showed that intensive glucose control in type 1 diabetes was associated with better cognitive test performance [93]. Similarly, the action to control cardiovascular risk in diabetes trial linked intensive glucose-lowering therapy to modest cognitive benefits in type 2 diabetes [94]. However, it is crucial to balance tight glycemic control with the risks of hypoglycemia, as recurrent low blood sugar episodes can adversely affect cognitive function. Severe hypoglycemic events have been associated with increased dementia risk and cognitive decline, potentially due to neuronal damage from glucose deprivation and excitotoxic neurotransmitter release [95].

**Table 1:** Summary of management strategies for cognitive impairment in diabetic patients

Management strategy	Description	References
Glycemic control	Optimizing blood glucose levels through lifestyle modifications, oral hypoglycemic agents, and/or insulin therapy	[91,92,93,94,95]
Lifestyle modifications	Diet: Adopting a balanced diet rich in fruits, vegetables, whole grains, lean proteins, and healthy fats; Mediterranean diet	[96,97,98,99]
	Physical activity: Regular aerobic exercise and resistance training to improve cognitive function	[100,101,102]
	Mind–body practices: Tai Chi, yoga, and other mind–body exercises	[103]
Cognitive training	Structured exercises to enhance specific cognitive domains, often delivered through computerized platforms or mobile apps	[104,105]
Pharmacological interventions	Cholinesterase inhibitors: Medications like donepezil and rivastigmine for memory and attention enhancement	[106]
	Antidiabetic drugs: Metformin and glucagon-like peptide-1 (GLP-1) agonists with potential neuroprotective properties	[107]
	Antioxidants and neuroprotective compounds: Resveratrol, curcumin, and omega-3 fatty acids (under investigation)	[108]
Multidisciplinary approach	Collaborative care involving endocrinologists, neurologists, dietitians, exercise physiologists, and other healthcare professionals	[109]
Early detection and intervention	Regular cognitive screening and monitoring integrated into routine diabetes care	[110,111]

#### 6.2.2 Lifestyle modifications

Adopting a balanced, healthy diet is crucial for managing cognitive decline in diabetics. Diets rich in fruits, vegetables, whole grains, lean proteins, and healthy fats have been linked to improved cognitive function and lower risk of decline [96]. The Mediterranean diet, high in plant-based foods, olive oil, and fish, shows potential in protecting brain health [97,98]. Specific components like omega-3 fatty acids may also benefit cognitive function [99]. Regular physical activity is consistently associated with improved cognitive outcomes and decreased decline risk in diabetics [100]. Exercise enhances glycemic control and insulin sensitivity, reduces inflammation, increases cerebral blood flow, and stimulates neurogenesis and brain plasticity [101]. Aerobic activities have shown effectiveness in boosting cognitive performance, particularly in executive function, memory, and processing speed [102]. Resistance training and mind-body practices may also enhance cognitive functions [103]. However, personalized workout regimens should be developed under healthcare provider guidance, considering individual factors such as age, health conditions, and physical limitations.

#### 6.2.3 Cognitive training

Cognitive training programs, consisting of structured exercises to enhance specific cognitive areas, are accessible through computerized platforms, mobile apps, or face-to-face sessions. Recent findings suggest these interventions could benefit individuals with diabetes-related cognitive impairment, especially when combined with lifestyle changes and medication [104]. These programs aim to boost cognitive reserve, stimulate neuroplasticity, and potentially slow or alleviate cognitive deterioration. Integrating lifestyle adjustments such as nutritious diet, regular physical activity, and cognitive training into comprehensive care plans for diabetics offers diverse advantages [105]. These interventions address underlying metabolic issues while enhancing brain health, potentially reducing cognitive deficits, and improving overall quality of life and well-being.

#### 6.2.4 Pharmacological interventions

While no food and drug administration-approved medications exist for treating diabetes-related cognitive impairment, various pharmacological agents have been explored. Cholinesterase inhibitors like donepezil and rivastigmine, originally for Alzheimer’s disease, have shown some memory and attention enhancements in diabetics with mild cognitive impairment [106]. Antidiabetic drugs such as metformin and GLP-1 agonists have demonstrated neuroprotective properties that could benefit cognitive function, though further studies are needed [107]. Antioxidants and neuroprotective compounds like resveratrol, curcumin, and omega-3 fatty acids are under investigation, but more extensive clinical trials are required to confirm their efficacy [108]. Other medications targeting specific mechanisms linked to cognitive impairment, including insulin sensitizers, anti-inflammatory agents, and drugs addressing vascular risk factors, are currently under scrutiny [104].

#### 6.2.5 Multidisciplinary approach and supportive care

The multifactorial nature of cognitive impairment in diabetes necessitates a collaborative, comprehensive approach involving various healthcare professionals. This team may include endocrinologists and primary care physicians managing glycemic control and complications, neurologists and neuropsychologists conducting cognitive assessments and developing personalized interventions [109]. Dietitians and exercise physiologists can guide healthy dietary patterns and tailored exercise programs. Social workers and support groups offer emotional support and resources, while occupational therapists assist with adaptive strategies and environmental modifications. This multidisciplinary approach, including ongoing monitoring and follow-up assessments, enhances overall management and support for individuals with diabetes-related cognitive impairment and their caregivers, improving well-being and quality of life. Healthcare professionals can track cognitive changes and adjust interventions as needed, providing comprehensive care for this complex condition.

## 7 Prevention and future directions

### 7.1 Importance of early detection and intervention

Early detection and intervention are crucial in mitigating cognitive impairment in diabetics. Cognitive deficits can be subtle and insidious, making early recognition challenging. Timely identification and intervention can potentially slow or reverse cognitive decline, preserve function, and improve quality of life. Regular cognitive screening and monitoring should be integrated into routine diabetes care, especially for those with known risk factors or comorbidities. Early detection facilitates prompt implementation of appropriate interventions, including lifestyle modifications, cognitive training, and potential pharmacological treatments [110]. Moreover, early intervention helps individuals and caregivers better understand and manage challenges associated with cognitive deficits, enabling the adoption of coping strategies and necessary adjustments to daily activities and routines [111].

### 7.2 Potential neuroprotective strategies

Current management of diabetes-related cognitive impairment focuses on modifiable risk factors and symptomatic relief. However, there is growing interest in neuroprotective strategies to potentially delay or halt cognitive decline, with various avenues under active exploration.

Targeting insulin resistance and insulin signaling: Targeting insulin resistance and signaling could yield neuroprotective benefits in diabetes-related cognitive impairment [112,113]. Insulin plays a critical role in preserving neurons, promoting synaptic plasticity, and supporting cognitive function [114]. Insulin resistance, characteristic of type 2 diabetes and a potential consequence of prolonged hyperglycemia, can disrupt brain insulin signaling, compromising neuronal function and cognition [115]. Ongoing research explores novel insulin sensitizers, compounds facilitating insulin transport across the BBB, and intranasal insulin formulations that could directly deliver insulin to the brain, potentially augmenting its neuroprotective properties [116].

Anti-inflammatory and antioxidative strategies: Oxidative stress and inflammation significantly contribute to neuronal damage and cognitive decline in diabetes [117]. Compounds with strong anti-inflammatory and antioxidative properties could potentially protect brain cells. Natural substances like resveratrol, curcumin, and omega-3 fatty acids are being investigated for their ability to alleviate oxidative stress, reduce brain inflammation, and enhance neuronal survival and adaptability [118,119]. Additionally, researchers are exploring synthetic compounds with antioxidative and anti-inflammatory qualities that target specific biological processes associated with cognitive decline [120].

Modulation of epigenetic mechanisms: Recent studies suggest that epigenetic changes, such as DNA methylation and histone modifications, play a crucial role in cognitive decline development. These alterations can modify gene expression patterns, leading to neuronal dysfunction and cognitive impairment [40,121]. Therapies or substances targeting these epigenetic pathways show potential for neuroprotection in diabetics [122]. By modulating gene expression patterns, these treatments could potentially restore normal neuronal function, support neuronal survival, and mitigate cognitive deficits.

Stem cell therapy and regenerative medicine: Research is exploring the potential of stem cells and regenerative medicine techniques to enhance neurogenesis, neural repair, and functional recovery in the diabetic brain [123,124]. Stem cell therapies could involve introducing exogenous stem cells or activating endogenous neural stem cells to replace damaged or dysfunctional neurons [125]. Scientists are also investigating the use of growth factors, small molecules, and other biomolecules to stimulate neurogenesis and promote neural repair and regeneration [126,127,128]. These strategies aim to restore the brain’s structural and functional integrity, potentially reversing or mitigating cognitive decline.

Lifestyle interventions and multimodal approaches: While pharmacological and regenerative medicine techniques show promise, holistic lifestyle interventions combining dietary changes, physical activity, cognitive exercises, and other strategies may offer synergistic neuroprotective benefits and improve overall brain health [129]. A comprehensive approach addressing multiple mechanisms and vulnerabilities could enhance the effectiveness of neuroprotective methods and improve cognitive outcomes for diabetics. Although many potential neuroprotective strategies are still in the early research stages, requiring thorough evaluation through rigorous clinical trials, their ongoing exploration holds promise for developing novel and effective interventions to combat diabetes-related cognitive impairment.

### 7.3 Areas for further research

Significant progress has been made in understanding the relationship between diabetes and cognitive impairment, but there are several areas that require further research. Priority should be given to elucidating the underlying molecular mechanisms linking diabetes with cognitive decline, including the interplay between hyperglycemia, insulin resistance, and vascular dysfunction [130]. Identifying reliable biomarkers and developing risk prediction models are crucial for early detection and timely intervention. Large-scale clinical trials are needed to evaluate the efficacy and safety of various interventions, encompassing pharmacological agents, cognitive training programs, and multimodal approaches. Longitudinal cohort studies tracking cognitive function, metabolic parameters, and biomarkers in diabetic patients from early disease stages are crucial for identifying early predictors and progression patterns of cognitive decline [131].

Advanced neuroimaging studies correlating brain structural and functional changes with cognitive performance over time can provide crucial insights into the underlying neurobiological mechanisms of diabetes-related cognitive decline. Complementing these findings, epigenetic and molecular studies are essential for investigating potential biomarkers and mechanisms, which can lead to the development of targeted interventions. To inform clinical decision-making and health policy, comparative effectiveness research on different pharmacological interventions, including cost-effectiveness analyses, should be conducted. The advent of big data and machine learning approaches offers promising avenues for identifying patterns and developing predictive models for early intervention. Ultimately, the integration of these research approaches can pave the way for personalized and precision medicine tailored to individual risk profiles, potentially leading to better outcomes for patients with diabetes at risk for cognitive decline [132].

## 8 Conclusion

Cognitive impairment is a prevalent complication of diabetes mellitus, affecting various cognitive domains. Its multifactorial pathophysiology involves chronic hyperglycemia, insulin resistance, and vascular factors. Risk factors include disease duration, poor glycemic control, diabetes complications, age, education level, comorbidities, and unhealthy lifestyle. Early detection and accurate diagnosis are crucial, utilizing cognitive screening tools, neuropsychological testing, and neuroimaging. Management strategies involve optimizing glycemic control, lifestyle modifications, pharmacological interventions, and a multidisciplinary approach. Current treatments primarily target modifiable risk factors and provide symptomatic relief. Ongoing research focuses on neuroprotective strategies, including targeting insulin resistance, exploring antioxidant and anti-inflammatory approaches, modulating epigenetic mechanisms, and leveraging regenerative medicine.

Recognizing cognitive impairment as a significant diabetes complication has important clinical implications. Routine cognitive screening and monitoring should be integrated into standard diabetes care, especially for high-risk individuals. Early detection enables prompt intervention. Healthcare professionals should adopt a holistic approach, addressing glycemic control and other modifiable risk factors. Multidisciplinary teams should collaborate to provide comprehensive assessment, personalized interventions, and continuous monitoring. Cognitive rehabilitation and training programs should be considered adjuncts to pharmacological and lifestyle interventions. Research efforts should continue to elucidate underlying mechanisms, identify biomarkers and risk prediction models, evaluate interventions through clinical trials, and explore personalized medicine approaches. Proactively addressing cognitive impairment in diabetics can improve outcomes, quality of life, and independence, reducing the burden on patients, caregivers, and healthcare systems.

## Abbreviations

AGEs: advanced glycation end-products
BBB: blood–brain barrier
GLP-1: glucagon-like peptide-1
ROS: reactive oxygen species
SES: socioeconomic status
